# The significance of autoantibodies to DFS70/LEDGFp75 in health and disease: integrating basic science with clinical understanding

**DOI:** 10.1007/s10238-015-0367-0

**Published:** 2015-06-19

**Authors:** Robert L. Ochs, Michael Mahler, Anamika Basu, Leslimar Rios-Colon, Tino W. Sanchez, Luis E. Andrade, Marvin J. Fritzler, Carlos A. Casiano

**Affiliations:** 1 Ventana Medical, Roche Tissue Diagnostics, Tucson, AZ USA; 2 Department of Research, Inova Diagnostics, Inc., San Diego, CA USA; 3 Department of Basic Sciences, Center for Health Disparities and Molecular Medicine, Loma Linda University School of Medicine, Mortensen Hall 142, 11085 Campus St, Loma Linda, CA 92350 USA; 4 Rheumatology Division, Universidade Federal de Sao Paulo, and Immunology Division, Fleury Medicine and Health Laboratories, São Paulo, Brazil; 5 Faculty of Medicine, University of Calgary, Calgary, AB Canada; 6 Department of Medicine, Division of Rheumatology, Loma Linda University School of Medicine, Loma Linda, CA USA

**Keywords:** Antinuclear autoantibodies, Autoimmunity, DFS70/LEDGFp75, Inflammation, Stress

## Abstract

Antinuclear autoantibodies (ANAs) displaying the nuclear dense fine speckled immunofluorescence (DFS-IIF) pattern in HEp-2 substrates are commonly observed in clinical laboratory referrals. They target the dense fine speckled autoantigen of 70 kD (DFS70), most commonly known as lens epithelium-derived growth factor p75 (LEDGFp75). Interesting features of these ANAs include their low frequency in patients with systemic autoimmune rheumatic diseases (SARD), elevated prevalence in apparently healthy individuals, IgG isotype, strong trend to occur as the only ANA specificity in serum, and occurrence in moderate to high titers. These autoantibodies have also been detected at varied frequencies in patients with diverse non-SARD inflammatory and malignant conditions such as atopic diseases, asthma, eye diseases, and prostate cancer. These observations have recently stimulated vigorous research on their clinical and biological significance. Some studies have suggested that they are natural, protective antibodies that could serve as biomarkers to exclude a SARD diagnosis. Other studies suggest that they might be pathogenic in certain contexts. The emerging role of DFS70/LEDGFp75 as a stress protein relevant to human acquired immunodeficiency syndrome, cancer, and inflammation also points to the possibility that these autoantibodies could be sensors of cellular stress and inflammation associated with environmental factors. In this comprehensive review, we integrate our current knowledge of the biology of DFS70/LEDGFp75 with the clinical understanding of its autoantibodies in the contexts of health and disease.

## Introduction

A hallmark of systemic autoimmune rheumatic diseases (SARD) such as systemic lupus erythematosus (SLE) and scleroderma is the presence of circulating, high-titer IgG autoantibodies targeting nuclear and cytoplasmic autoantigens of protein or nucleic acid nature [1]. These “antinuclear autoantibodies” (ANAs), are typically detected by indirect immunofluorescence (IIF) microscopy in commercially available HEp-2 ANA test slides and have been extensively used as biomarkers in the differential diagnosis of SARD and molecular probes for the discovery and characterization of novel intracellular autoantigens [1]. They can also be detected in non-SARD conditions such as cancer and are considered as “messengers” or “reporters” of molecular and cellular events that induce an autoimmune response [1, 2].

Autoantibodies targeting the nuclear autoantigen DFS70/LEDGFp75 have attracted much interest given their relatively common occurrence in patient sera referred to clinical laboratories for ANA-HEp-2 testing [3–7]. While DFS70/LEDGFp75 has emerged as a multifunctional stress response protein of high relevance to acquired immunodeficiency syndrome (AIDS), cancer, inflammation and other human conditions [8–12], several unanswered questions concerning the clinical and biological significance of its associated autoantibodies still remain. Why are high-titer anti-DFS70/LEDGFp75 autoantibodies common among patients with positive ANA tests who are asymptomatic for SARD? Are there differences in the frequencies and clinical associations of these autoantibodies in young versus older people? What makes DFS70/LEDGFp75 immunogenic in some apparently healthy individuals (HI) and patients with non-SARD inflammatory conditions? Are these antibodies protective, pathogenic, or sensors of underlying inflammatory pathologies? Do all human sera positive for autoantibodies recognizing the nuclear dense fine speckled immunofluorescence pattern (DFS-IIF) specifically target DFS70/LEDGFp75? In the following sections, we address these questions while integrating our basic and clinical knowledge of this autoantigen-autoantibody system.

## Discovery of DFS70/LEDGFp75

A timeline of key milestones in the discovery and characterization of the DFS70/LEDGFp75 autoantigen-autoantibody system is presented in Table 1. The DFS70 autoantigen was originally identified in the 1990s during surveys of ANAs in patients with interstitial cystitis (IC) and chronic fatigue syndrome (CSF) [3, 4]. Using a high-titer serum from an IC patient producing a strong DFS-IIF pattern, a complementary DNA expression library was screened and a partial DNA sequence for DFS70 was obtained [3]. This sequence was deposited in GenBank in 1997, and no other sequence match was detected at the time [3]. When the complete DFS70 sequence was later entered into GenBank, it was found to be identical to a newly discovered gene named transcription coactivator p75 (TCp75) and LEDGFp75 [3, 13, 14]. TCp75 and its shorter splicing variant p52 were identified as transcription coactivators of the RNA polymerase II complex [13], whereas LEDGFp75 was identified as a lens epithelium cell (LEC)-derived autoantigen targeted by autoantibodies in a patient with cataracts [9, 14]. Initial studies suggested that LEDGFp75 was a growth factor in LECs [9, 14, 15]; however, it is now recognized that this protein is ubiquitously present in mammalian cells, playing roles more consistent with stress protection than growth factor function. The gene encoding this autoantigen is also designated *PSIP1* (PC4 and SFRS1 interacting protein 1) [16], although the names DFS70 and LEDGFp75 are the most commonly used for the protein. Following the initial discovery of DFS70/LEDGFp75, three independent groups made the seminal discovery that this protein is a key cellular co-factor for HIV-1 integration into host chromatin [17–20].

**Table 1:** Key milestones in the history of the DFS70/LEDGFp75 autoantigen-autoantibody system

Year	Milestone	References
1994	Discovery of serum autoantibodies recognizing the nuclear DFS-IIF pattern in patients with interstitial cystitis	[4]
1997	Partial cDNA sequence encoding the autoepitope region of the DFS70 autoantigen deposited in GenBank under accession number U94319	[3]
1998	Discovery of transcription co-activator p75, later known to be identical to DFS70 and LEDGFp75	[13]
1999	Discovery of LEDGFp75 using autoantibodies from a cataract patient	[14]
2000	Characterization of DFS70 using autoantibodies from patients with atopic dermatitis and other conditions, and initial observation that these autoantibodies are present at low frequencies in SARD patients	[3]
2001–2002	Demonstration that DFS70/LEDGFp75 is cleaved during cell death into fragments that are recognized by autoantibodies	[32, 98]
2003–2004	Discovery of DFS70/LEDGFp75 as a key cellular co-factor of HIV-1 integration	[17–20]
2004	Initial observation that anti-DFS70/LEDGFp75 autoantibodies are present in apparently healthy individuals	[125]
2004	Identification of a major B cell autoepitope in the carboxy-terminal region of DFS70/LEDGFp75	[62]
2005	Identification of DFS70/LEDGFp75 as a tumor associated autoantigen	[99]
2005	Demonstration that anti-DFS70/LEDGFp75 autoantibodies are a very common occurrence in human sera screened for ANAs by HEp-2 IIF in a clinical laboratory and can be detected in a wide array of immunological conditions	[7]
2008	Observation that patients producing anti-DFS70/LEDGFp75 autoantibodies as the only serum ANA pattern are rarely diagnosed with SARD	[28]
2011–2012	Description of anti-DFS70/LEDGFp75 autoantibodies as a potential exclusion biomarker for SARD	[26, 107]
2012	Development of a highly specific ANA test based on immunoadsorption of anti-DFS70/LEDGFp75 autoantibodies	[5]
2012	Introduction of a new algorithm for ANA testing that considers anti-DFS70/LEDGFp75 autoantibodies	[6]
2013	First commercially available diagnostic test (Inova Diagnostics) for the detection of anti-DFS70/LEDGFp75 antibodies	N/A

## General properties of anti-DFS70/LEDGFp75 autoantibodies

**Fig. 1:**
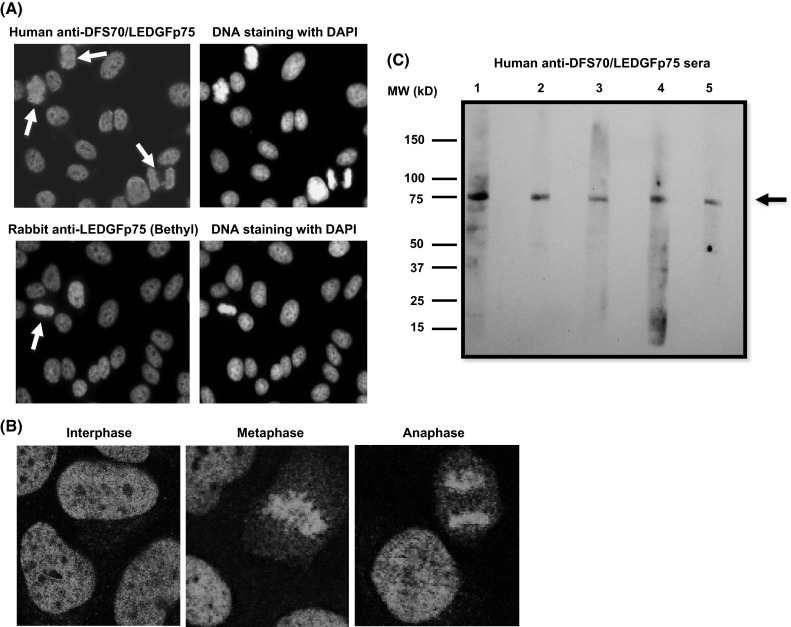
Characteristic features of human autoantibodies to DFS70/LEDGFp75. **a** Staining pattern produced by human and rabbit autoantibodies to DFS70/LEDGFp75 in HEp-2 slides visualized by IIF microscopy using FITC-labeled secondary antibodies. *Yellow arrows* point to bright staining in condensed metaphase chromosomes. **b** Confocal microscopy images showing reactivity of a human DFS70/LEDGFp75 autoantibody in U2OS cells. **c** Immunoblot showing reactivity of representative DFS-IIF-positive patient sera against a single band of approximately 75 kD in PC3 cell lysates (Color figure online)

These autoantibodies are predominantly IgG, often reaching high titers in healthy individuals and patients with diverse inflammatory diseases [3, 21–26]. They recognize a protein of 70–75 kD on immunoblots (predicted molecular size of 60 kD) that can be visualized by IIF microscopy as dense fine speckles in the nucleoplasm of cells in interphase, typically excluding the nucleolus, with increased staining intensity of condensed mitotic chromosomes [3–6] (Fig. 1). Muro and colleagues observed that very few patients with SARD produce these antibodies, and usually in combination with other SARD-marker autoantibodies such as anti-DNA, anti-p80 coilin, and anti-topo I [27, 28]. They also showed increased frequencies of human leukocyte antigen (HLA)-DRB1, (HLA)-DQB1, and (HLA)-DPB1 alleles in patients with anti-DFS70/LEDGFp75 antibodies, although a strong correlation between these autoantibodies and specific HLA alleles could not be established [29].

## DFS70/LEDGFp75 structure and function

### Gene and spliced variants

**Fig. 2:**
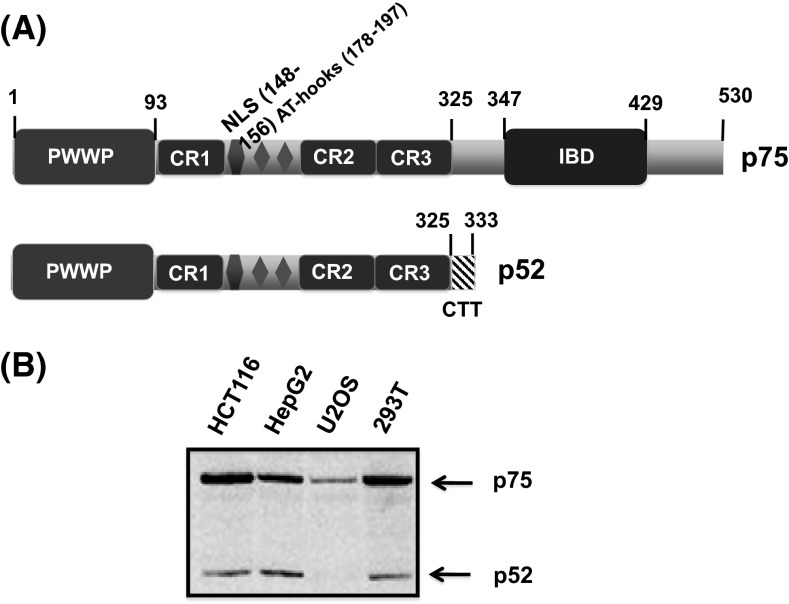
Main splicing variants of DFS70/LEDGFp75. **a** Depiction of the two major splice variants of DFS70/LEDGFp75, namely p75 and p52, with their domains and motifs. **b** Immunoblot showing the reactivity of a commercial monoclonal antibody (BD Biosciences) directed against the N-terminal region of DFS70/LEDGFp75 that recognizes both splice variants in a panel of cancer cell lines

DFS70/LEDGFp75 and its short splice variant LEDGF/p52 (hereafter referred to as p52) (Fig. 2a) are derived from the same *PS1P1/LEDGF* gene, which consists of 15 exons and 14 introns, with exons 1–15 encoding DFS70/LEDGFp75, and exons 1–9 and a small part of intron 9 (24 nucleotides) encoding p52 [30]. Although other alternatively spliced variants of this gene have been identified [31], DFS70/LEDGFp75 and p52 are the most common based on immunoblotting analysis of cell lysates (Fig. 2b) [32–34]. DFS70/LEDGFp75 and p52 share an amino (N)-terminal region (residues 1–325); however, p52 has an intron-derived C-terminal tail (CTT, residues 326–333) that is not present in DFS70/LEDGFp75 (Fig. 2a). These variants localize to different nuclear regions and appear to play opposing roles when ectopically overexpressed, with DFS70/LEDGFp75 acting as a stress survival protein and p52 as a pro-apoptotic protein [33, 35]. P52 has been particularly implicated in splicing regulation through binding to trimethylated histone H3K36me3 and splicing factor SRSF1, and in the regulation of neurite growth in rat retinal ganglion cells [36–39].

### Structural and functional domains

The N-terminal region shared by DFS70/LEDGFp75 and p52 contains a PWWP domain (Fig. 2a), defined by a proline-tryptophan-tryptophan-proline motif (residues 19–22). PWWP domains are highly conserved in members of the hepatoma-derived growth factor (HDGF) family and in several DNA-binding proteins and have been implicated in chromatin binding, HIV-integration, protein–protein interactions, transcription, and DNA methylation [40–43]. This domain facilitates the dynamic scanning and hopping of DFS70/LEDGFp75 along the chromatin, and the locking into chromatin of interacting proteins that are bound to its C-terminus [44]. Binding of this domain to chromatin is facilitated by its interaction with H3K36me3 [45]. Other sequences such as positively charged regions, a nuclear localization signal and AT-hook motifs (Fig. 2a), also contribute to DFS70/LEDGFp75 binding to chromatin, particularly to H3K4me3 at active transcription sites [46–50].

Both the N- and C-terminal regions of DFS70/LEDGFp75 contribute to its transcription and stress survival functions by engaging in interactions with chromatin-binding proteins, or by binding to promoters of specific stress genes [33, 42, 48, 51–56]. Chromosomal translocations in leukemia produce a NUP98-LEDGFp75 fusion protein lacking the N-terminal region of DFS70/LEDGFp75, resulting in a PWWP-deficient protein with deregulated transcription functions [56–60]. The C-terminal region of DFS70/LEDGFp75 (residues 325–530), absent in p52, contains two helix-turn-helix domains (residues 421–442 and 471–492) that are capable of binding heat shock elements within promoter regions of stress genes [48]. The C-terminal region of DFS70/LEDGFp75 also encompasses the HIV integrase-binding domain (IBD, residues 347–429), which is recognized by the HIV-1 integrase (HIV-IN) [17–20, 61].

### Autoepitope mapping in DFS70/LEDGFp75

The C-terminus of DFS70/LEDGFp75 contains the autoepitope (aa 347–429) recognized by the autoantibodies [62], which explains why these consistently recognize a single band of 70–75 kD and not the p52 variant in immunoblots of cell lysates (Fig. 1b). Intriguingly, this immunogenic region is essentially the same region comprised by the IBD [18, 61, 63] and shares significant homology with HRP-2 (HDGF-related protein 2), a member of the HDGF protein family that can also interact with HIV-IN [64].

The biological significance of these coincidental findings is not presently clear and raises interesting questions. Why would an epitope region targeted by autoantibodies correspond to the exact same region specifically recognized by the HIV-IN? What structural or functional elements within this region make it attractive for targeting by both the immune system and the HIV-1 virus? While at the present time we lack sufficient information to answer these questions, it is evident that the autoepitope/IBD region has intrinsically important cellular functions. These include, in addition to HIV-IN binding, serving as a hub for protein–protein interactions in the chromatin, regulation of gene expression, and stress survival activity. It should be noted that DFS70/LEDGFp75 is predicted to be a highly disordered protein, a feature of proteins that have multiple interacting partners [61]. One could speculate that the largely disordered nature of DFS70/LEDGFp75 and the promiscuity of its autoepitope/IBD domain in interacting with multiple proteins (both self and non-self), may influence its proteolytic processing and presentation to the immune system. This could contribute to enhanced immunogenicity under pro-inflammatory conditions, leading to autoantibody generation in susceptible individuals. The autoimmune targeting of the IBD is consistent with the notion that autoantibody-defined epitopes typically comprise highly conserved, conformational, and functional domains [1, 2].

## Cellular and tissue expression of DFS70/LEDGFp75

DFS70/LEDGFp75 exhibits transcript expression in various human tissues [14]. Its expression may be differentiation-related, as suggested by the higher mRNA and protein levels in fetal brain compared to adult brain, and its loss from nuclei of differentiating LECs [65, 66]. DFS70/LEDGFp75 is also expressed in LECs, keratinocytes, fibroblasts, and most laboratory transformed cell lines, with elevated levels in cancer cells [14, 15, 31–34].

## Cellular functions of DFS70/LEDGFp75

### Protection against environmental stress

Compelling evidence supports a cellular protective function for DFS70/LEDGFp75 against environmental factors that induce cellular stress, such as ultraviolet B (UVB) irradiation, hydrogen peroxide, alcohol, hyperthermia, nutrient deprivation, and certain chemotherapeutic drugs [14, 31, 32, 34, 51, 67–73]. These stressors can lead to increased oxidative stress, which induces upregulation and activation of DFS70/LEDGFp75 [73].

DFS70/LEDGFp75 is presumed to promote cellular protection against environmental stressors by transcriptionally activating stress, antioxidant, and other protective genes [51, 74–82]. However, to date only a few target genes of DFS70/LEDGFp75 have been identified and validated (Table 2). While global gene profiling studies on cells stably depleted of DFS70/LEDGFp75 failed to reveal a specific genetic pathway regulated by this protein [12], studies using pathway-specific gene arrays showed that ectopic overexpression or transient depletion of this protein in cancer cells under stress led to significant changes in the expression of certain stress and antioxidant genes [82]. These findings suggested that DFS70/LEDGFp75 contributes to the regulation of stress gene expression mainly when it is upregulated and activated under stress.

**Table 2:** List of candidate target genes of DFS70/LEDGFp75

Gene	Description	Method of discovery	Validation	References
ADH and	Alcohol dehydrogenase and	EMSA	Transcription reporter assays	[78]
ALDH	aldehyde dehydrogenase			
ALB	Albumin	qPCR Array RNAi, overexpression	qPCR	[82]
AOP2/PRDX6	Antioxidant protein 2/Peroxiredoxin 6	DNase I footprinting, EMSA	Transcription reporter assays, qPCR, immunoblotting	[74]
CYGB	Cytoglobin	qPCR Array RNAi, overexpression	qPCR, immunoblotting	[82]
HOX genes	Homeobox genes	Gene microarray, RNAi	qPCR	[16, 56, 85]
HSP27	Heat shock protein 27	DNase I footprinting	Transcription reporter assays, RT-PCR, RNAi	[42, 51, 87]
IL-6	Interleukin 6 (interferon, beta 2)	qPCR, immunoblotting, overexpression	RNAi	[103, 104]
INV	Involucrin	EMSA	Transcription reporter assays, qPCR, immunoblotting, IHC	[75]
PIP3-E/IPCEF-1	Phosphoinositide-binding protein/Interacting protein for cytohesin exchange factor 1	RT-Profiler qPCR Array RNAi, overexpression	qPCR	[82]
SOD3	Superoxide dismutase 3	qPCR Array, RNAi, overexpression	qPCR	[82]
TPO	Thyroid peroxidase	qPCR Array, RNAi, overexpression	qPCR	[82]
VEGF-C	Vascular endothelial growth factor C	ChIP	RT-PCR, immunoblotting, transcription reporter assays	[80, 81]
αB crystallin	Small stress protein alpha basic crystallin	DNase I footprinting	Transcription reporter assays, RT-PCR, EMSA, RNAi	[51, 79]
γ-GCS-HS	Gamma glutamyl cysteine synthetase-heavy subunit	Transcription reporter assays	qPCR, immunoblotting, RNAi	[71]

*ChIP* chromatin immunoprecipitation, *EMSA* electrophoretic mobility shift assay, *IHC* immunohistochemistry, *RNAi* RNA interference, *RT-PCR* reverse transcription polymerase chain reaction, *qPCR* quantitative, real-time PCR

### DFS70/LEDGFp75 interactome

Both the PWWP domain and the C-terminal IBD region of DFS70/LEDGFp75 interact with various chromatin-associated proteins, likely facilitating DFS70/LEDGFp75 function in stress gene expression regulation. Interactors of the IBD in addition to HIV-IN include the pogo transposable element PogZ, the c-Myc interacting protein JPO2, the Cdc7-activator of S-phase kinase (ASK), and the leukemia-associated transcription complex Menin-MLL (mixed lineage leukemia) [52–56, 83]. The PWWP domain has been implicated in binding to the methylation-associated protein MeCP2, transcription coactivator TOX4, and splicing cofactor NOVA1 [42, 84]. DFS70/LEDGFp75 also participates in the recruitment of polycomb group protein Bmi1 and co-repressor Ctbp1 to MLL complexes in *HOX* gene promoters [85].

### Cell death and survival decisions

Various groups have reported that depletion or functional inactivation of DFS70/LEDGFp75 leads to decreased cell survival [15, 32, 33, 57, 72, 86, 87]. However, these results are controversial because others have reported that this protein is not essential for cell survival based on observations that cell clones with stable depletion of the protein can survive in culture [88, 89]. In addition, a *PSIP1/LEDGF*^*−/*−^ knockout mouse model revealed that disruption of this gene is not intrinsically lethal to mice [16]. While these conflicting observations are likely to be cell type- and context-dependent, it is possible that selected cell clones with stable depletion of DFS70/LEDGFp75 may have developed compensatory mechanisms to survive in the absence of this protein. This is supported by the observation that prostate cancer (PCa) cell clones with stable DFS70/LEDGFp75 depletion did not display significant changes in stress gene expression when compared to stressed cells with transient depletion [82].

The possibility that DFS70/LEDGFp75 is needed mainly in the context of stress survival was suggested by the observation that deletion mutants of the protein lacking portions of its N- and C-terminal domains did not show any effects on cell death or survival when stably overexpressed in cancer cells growing under normal conditions [32]. However, unlike the full-length protein, these mutants were unable to support cell survival under starvation stress [32].

Transactivation of protective genes by DFS70/LEDGFp75 under cellular stress is likely to contribute to preservation of the structural integrity of critical organelles that are highly susceptible to oxidative damage and that regulate cell death and survival. Consistent with this, DFS70/LEDGFp75 was shown to protect cancer cells against antitumor drugs that induce lysosomal membrane permeabilization (LMP) and cell death [34, 72]. PCa cell lines selected in culture for natural resistance to docetaxel, an antitumor drug that induces LMP and is antagonized by DFS70/LEDGFp75, express high levels of this autoantigen, consistent with the possibility that chemotherapeutic stress induces its expression [31, 34, 82].

DFS70/LEDGFp75 has also been implicated in cellular protection against oxidative DNA damage. It enhanced the survival of retinal pigment epithelial cells challenged by oxidative stress or UVB irradiation [70], a survival effect associated with DFS70/LEDGFp75-mediated protection of DNA from single-strand breakage and upregulation of Hsp27. This is consistent with studies showing that DFS70/LEDGFp75 promotes repair of DNA double-strand breaks through the homologous recombination repair pathway [90].

## Regulation of DFS70/LEDGFp75 expression and function

### Transcriptional regulation

Increased cellular expression of the Sp1 transcription factor leads to upregulation of DFS70/LEDGFp75 via TATA-less promoter activation, while its inhibition represses this upregulation [91, 92]. However, during LEC exposure to UVB, a histone deacetylase/histone methylase (HDAC1/SUV39H1) complex is recruited to Sp1 responsive elements in the DFS70/LEDGFp75 gene promoter, leading to attenuation of Sp1 binding, repression of DFS70/LEDGFp75 expression, and increased cellular oxidative stress and death [93]. These results suggest that certain stressors may either upregulate or repress DFS70/LEDGFp75 depending on context.

Transforming growth factor beta 1 (TGF-β1) is also known to downregulate DFS70/LEDGFp75 in LECs by binding to its promoter region [77]. This is consistent with the observations that a *Prdx6*^−*/*−^ knockout mouse cell line displayed increased TGF-β1 levels with reduced DFS70/LEDGFp75 [94] and that an inverse expression relationship between these genes exists in diabetic and galactosemic cataractous rat lenses [95].

There is also evidence that DFS70/LEDGFp75 is regulated at the transcriptional level by micro-RNAs (miRNAs). Macrophages stimulated with lipopolysaccharide induced miR-155, concomitant with downregulation of DFS70/LEDGFp75, and ectopic expression of this miRNA reduced DFS70/LEDGFp75 expression at the transcriptional level [96]. Another miRNA, miR-135b, also downregulated DFS70/LEDGFp75 both in human cell lines and in murine vestibular sensory epithelia of the inner ear, and it was suggested that this downregulation could influence inner ear cell survival, protection against stress, development, and differentiation [97].

### Functional regulation by repression of transcription function

**Fig. 3:**
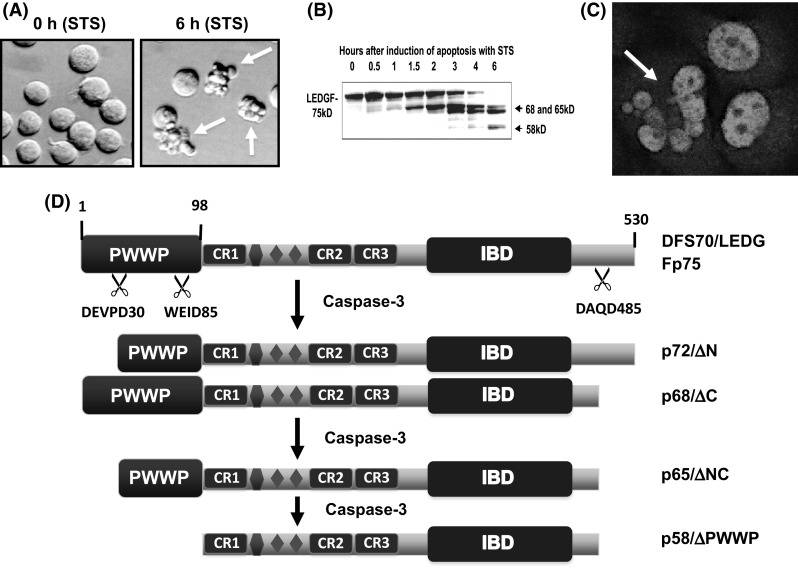
Apoptotic cleavage of DFS70/LEDGFp75. **a** Jurkat T cells undergoing apoptosis after exposure to staurosporine (STS). **b** Immunoblot showing cleavage of DFS70/LEDGFp75 into various fragments during STS-induced apoptosis in Jurkat cells. These fragments were detected with human autoantibodies to DFS70/LEDGFp75. **c** Confocal microscopic image of DFS70/LEDGFp75 autoantibody staining in U2OS cells undergoing apoptosis (*yellow arrow* depicts apoptotic blebs retaining DFS70/LEDGFp75 staining). **d** Diagram illustrating caspase-mediated sequential cleavage of DFS70/LEDGFp75 (Color figure online)

Differential expression of DFS70/LEDGFp75 and its short splice variant p52 was observed in a panel of cancer cell lines, with DFS70/LEDGFp75 expressed at higher levels [32–34]. Interestingly, ectopic overexpression of p52 induced decreased cell survival via caspase-dependent apoptosis associated with DFS70/LEDGFp75 cleavage [33]. During apoptosis, caspase-3 cleaves DFS70/LEDGFp75 to generate several fragments [32, 33, 98, 99]. As shown in Fig. 3, treatment of Jurkat T cells for 6 h with staurosporine (STS) induces the classical apoptosis morphology (Fig. 3a), which is associated with cleavage of DFS70/LEDGFp75 into fragments of 68, 65, and 58 kD that are recognized by the autoantibodies in immunoblots of whole-cell lysates (Fig. 3b). Consistent with this, autoantibody recognition of DFS70/LEDGFp75 in apoptotic blebs can be detected by IIF microscopy (Fig. 3c). These fragments are produced by caspase-3-mediated sequential cleavage of the protein at specific aspartic acid residues located in the N-terminal PWWP domain and the C-terminal region (Fig. 3d). This apoptotic cleavage impairs DFS70/LEDGFp75’s stress survival activity and generates fragments that enhance cell death under stress [32]. An interesting observation was that during apoptosis, p52 is also cleaved by caspase-3 to generate a p38 fragment that antagonizes the transcriptional function of DFS70/LEDGFp75 [33].

The pro-survival protein Bcl-2 was also shown to attenuate DFS70/LEDGFp75 stress survival and transcriptional activities in LECs by interfering with its binding to the promoter region of the gene encoding αB crystallin [100]. It is plausible that these pro-survival proteins antagonize different cell death pathways, prompting cells to tightly regulate their expression.

### Functional regulation by sumoylation

DFS70/LEDGFp75 is post-translationally sumoylated at different sites in its N-terminal and C-terminal regions [101]. While sumoylation does not affect its cellular localization and chromatin-binding ability, it provides a mechanism to regulate its transcriptional activity, as suggested by the observation that mutations of DFS70/LEDGFp75 sumoylated sites extended its half-life and increased its transcriptional activity [101]. Consistent with these observations, LECs expressing sumoylation-deficient DFS70/LEDGFp75 displayed increased transcriptional and cellular survival activities compared to wild-type protein [102]. These results suggested that sumoylation of this autoantigen is a mechanism to regulate stress responses.

### Crosstalk between DFS70/LEDGFp75 and inflammatory pathways

Overexpression of DFS70/LEDGFp75 has been shown to induce the IL-6/STAT3 signaling pathway in HaCaT skin cells, whereas its knockdown reduced IL-6 levels [103, 104]. Interestingly, the intracellular localization of both DFS70/LEDGFp75 and phosphorylated STAT3 in HaCaT cells appears to be regulated by Ran-binding proteins, which suggested that a similar mechanism may be operating in psoriatic keratinocytes [105]. Additional evidence supporting a link between DFS70/LEDGFp75 and STAT3 came from studies demonstrating that switching the expression of STAT3 to STAT3β, its dominant negative truncated variant, in cancer cells led to DFS70/LEDGFp75 repression [106]. These observations suggested that DFS70/LEDGFp75 is activated by STAT3 in an autocrine/paracrine loop and raised the interesting possibility that a regulatory crosstalk between this autoantigen and the IL-6/STAT3 pathway may contribute to inflammatory processes [106]. Consistent with a link between DFS70/LEDGFp75 activation and inflammation, Takeichi et al. [104] also observed that DFS70/LEDGFp75 stimulated release of the cytokines TNF and IL-8 from keratinocytes. Interestingly, treatment of LECs with sublethal doses of TNF resulted in induction of oxidative stress and elevated expression of DFS70/LEDGFp75, which in turn transactivated the protective protein gamma glutamylcysteine synthetase [71].

## Clinical associations of anti-DFS70/LEDGFp75 autoantibodies

During the past 15 years, several groups have documented the presence of autoantibodies to DFS70/LEDGFp75 in apparently healthy individuals (HI) and in a variety of conditions associated with inflammation and oxidative stress (Table 3). Below, we briefly discuss these clinical associations and their implications.

**Table 3:** Clinical associations of autoantibodies to DFS70/LEDGFp75 or sera presenting the dense fine speckled pattern

Category	Reactivity (positive/total)	Frequency (%)	Detection methods	References
Alopecia areata	22/111	20	HEp-2 IIF, WB, ELISA	[21]
Arthralgia	16 of 81 DFS-positive sera	19.8	HEp-2 IIF	[7]
	2 of 34 DFS-positive sera	5	HEp-2 IIF, CIA	[130]
Asthma	8/50	16	HEp-2 IIF, WB	[3]
	1/25	4	CIA	[107]
Atopic dermatitis	19/64	29.6	HEp-2 IIF, WB,	[3]
	15/21 (children)	71.4	ELISA	[108]
	23/61	37.7	ELISA	[23]
	0/16	0	CIA	[107]
Atopic dermatitis with cataract	8/8	100	ELISA	[108]
Atypical retinal degeneration	ND (3 case studies)	NA	WB, SEREX	[110]
Autoimmune fatigue syndrome	226 cases, reactivity not clearly stated	~40	ELISA, WB	[115]
Autoimmune thyroiditis	13 of 81 DFS-positive sera	16	HEp-2 IIF	[7]
	4/67	6	CIA	[107]
Behcet’s disease	11/32	34.4	ELISA	[25]
Blood bank donors	35/650	5.4	ELISA	[108]
Cancer (various types)	6/334	1.8	HEp-2 IIF	[22]
	0/40	0	HEp-2 IIF, CIA	[107]
	3 of 81 DFS-positive sera	3.7	HEP-2 IIF	[7]
Clinical referrals or routine	53/3263	1.6	HEp-2 IIF, ELISA, CIA	[107]
ANA testing	5,081 of 13,641 ANA-positive sera	37	HEp-2 IIF	[7]
	172/21,516	0.8	HEp-2 IIF	[22]
	101/2,654	3.8	HEp-2 IIF	[128]
	101 of 352 ANA-positive sera	28.7	HEp-2 IIF	[128]
	57/2,788	2	HEp-2 IIF	[129]
	57 of 790 ANA-positive sera	7.2	HEp-2 IIF	[129]
Chronic fatigue syndrome	2/60	3.3	HEp-2 IIF, WB	[3]
	2 of 81 DFS-positive sera	2.5	HEp-2 IIF	[7]
	18 of 21 ANA-positive children	86	HEp-2 IIF, WB	[116]
	36 cases, reactivity not clearly stated	~40	ELISA, WB	[115]
Diverse dermatological conditions	39 of 115 ANA-positive sera	34	HEp-2 IIF	[128]
Dermatomyositis/polymyositis	4/80	5	HEp-2 IIF, WB	[28]
	7/116	6	HEp-2 IIF, ELISA	[123]
Diffuse pain	21 of 81 DFS-positive sera	26	HEp-2 IIF	[7]
Fibromyalgia	3 of 81 DFS-positive sera	3.7	HEp-2 IIF	[7]
	3 of 34 DFS-positive sera	9	HEp-2 IIF, CIA	[130]
	1 of 15 ANA-positive children	4.8	HEp-2 IIF, WB	[116]
Graves disease	1/60	1.7	CIA	[107]
Gynecologic syndromes	2 of 81 DFS-positive sera	2.5	HEp-2 IIF	[7]
Healthy donors	0/39	0	HEp-2 IIF, WB	[3]
	8/37	21.6	ELISA	[25]
	64/597	11	HEp-2 IIF, WB, ELISA	[125]
	8/105	7.6	WB, ELISA	[21]
	11/124	8.9	HEp-2 IIF, ELISA,	[107]
	39/918	4.2	CIA HEp-2 IIF	[26]
	39 of 118 ANA-positive sera	33.1	HEp-2 IIF	[26]
	16 of 34 DFS-positive sera	47	HEp-2 IIF, CIA	[130]
Infectious diseases	0/20	0	CIA	[107]
	6 of 81 DFS-positive sera	7.4	HEp-2 IIF	[7]
Inflammatory bowel disease	0/34	0	CIA	[107]
Interstitial cystitis	9/103	8.7	HEp-2 IIF, WB	[3]
	2/40	5	CIA	[107]
Multiple sclerosis	0/10	0	CIA	[107]
	1 of 81 DFS-positive sera	1.2	HEp-2 IIF	[7]
	2 of 172 DFS-positive sera	1.2	HEp-2 IIF	[22]
Prostate cancer (PCa)	46/206	22.3	HEp-2 IIF, ELISA, WB	[99]
Matched controls for PCa	9/164	5.4	ELISA	[99]
Rheumatoid arthritis	0/30	0	HEp-2 IIF, WB,	[3]
	0/40	0	HEp-2 IIF, WB, ELISA	[125]
	16 of 172 DFS-positive sera	9.3	HEp-2 IIF	[22]
	1/39	2.6	CIA	[107]
	2 of 81 DFS-positive sera	2.4	HEp-2 IIF	[7]
	0/13	0	HEp-2 IIF, WB	[28]
	11/65	16.9	HEp-2 IIF	[128]
	2/13	15.3	HEp-2 IIF	[128]
	2 of 34 DFS-positive sera	5.8	HEp-2 IIF, CIA	[130]
Sarcoidosis	4/16	25	ELISA	[25]
Scleroderma/systemic sclerosis	1/40	2.5	HEp-2 IIF, WB	[3]
	0/50	0	HEp-2 IIF, WB, ELISA	[125]
	2 of 172 DFS-positive sera	1.2	HEp-2 IIF	[22]
	0/29	0	CIA	[107]
	1/164	0.006	HEp-2 IIF, WB	[28]
	1 of 91 DFS-positive sera	1.1	CIA	[24]
Sjögren’s syndrome	2/29	6.9	HEp-2 IIF, WB	[3]
	2/30	6.7	HEp-2 IIF, WB, ELISA	[125]
	4 of 172 DFS-positive sera	2.3	HEp-2 IIF	[22]
	0/7	0	CIA	[107]
	8/71	11.3	HEp-2 IIF, WB	[28]
	2 of 34 DFS-positive sera	5.8	HEp-2 IIF, CIA	[130]
Systemic lupus erythematosus	0/36	0	HEp-2 IIF, WB	[3]
	1/55	2	HEp-2 IIF, WB, ELISA	[125]
	5 of 172 DFS-positive sera	2.9	HEp-2 IIF	[22]
	7/251	2.8	CIA	[107]
	5 of 81 DFS-positive sera	6.2	HEp-2 IIF	[7]
	7/124	5.6	HEp-2 IIF, WB	[28]
	4 of 91 DFS-positive sera	4.3	CIA	[24]
Sympathetic ophthalmia	5/7	71.4	ELISA	[25]
Vogt–Koyanagi–Harada Syndrome	24/36	66.7	ELISA	[25]

*CIA* Inova QuantaFlash chemiluminescence assay, *DFS* dense fine speckled, *ELISA* enzyme-linked immunosorbent assay, *IIF* indirect immunofluorescence microscopy, *WB* Western blotting, *IP* immunoprecipitation, *ND* not determined, *NA* not available

### Autoantibodies to DFS70/LEDGFp75 in IC

Ochs et al. [3, 4] investigated the presence of ANAs in sera of IC patients, especially noting whether or not their specificities were unique or similar to SARD-related ANAs. Among the ANA patterns observed in sera from 96 patients, there was a predominance of the nuclear DFS-IIF pattern (69 % of all ANA-positive patients and 9 % of the total cohort). Immunoblotting analysis confirmed that these DFS-positive sera contained autoantibodies to DFS70/LEDGFp75 [3]. A later study found only a 5 % frequency of these antibodies in IC patients when detected by the Quanta Flash DFS70 chemiluminescence assay (DFS70-CIA) [107].

### Anti-DFS70/LEDGFp75 autoantibodies in atopic and skin disorders

Ochs et al. [3] also observed that sera from 28 to 16 % patients with atopic dermatitis (AD) and asthma, respectively, produced the DFS-IIF pattern. Immunoblotting with recombinant DFS70/LEDGFp75 confirmed the presence of IgG and IgE autoantibodies to this protein. Other disease cohorts tested, including SARD, revealed low frequency (<5 %) of these antibodies, suggesting that this autoantibody-autoantigen system is not associated with SARD [3].

The presence of anti-DFS70/LEDGFp75 autoantibodies in AD was confirmed by the observation that some AD patients producing these autoantibodies also had cataracts [108]. In addition, these antibodies (both IgE and IgG4) were detected at a prevalence of 15 % in AD patients, and their presence correlated with elevated thymus and activation-regulated chemokine, which is associated with increased AD severity [23]. Immunohistochemical (IHC) analysis showed that DFS70/LEDGFp75 was present in epidermal cells and infiltrating monocytes in the skin of AD patients, suggesting that DFS70/LEDGFp75 upregulation or release from damaged tissue or invading cells may trigger autoantibody responses [23]. It should be noted, however, that the elevated prevalence of these autoantibodies in AD could not be confirmed using DFS70-CIA, highlighting the inter-laboratory and inter-diagnostic platform differences in the detection of these autoantibodies [107]. Differences in cohort collection and composition (age, gender, race) may also explain these discrepancies.

DFS70/LEDGFp75 is predominantly located in the nucleus of basal epidermal cells and then translocates into the cytoplasm during differentiation, where it accumulates in the granular layer of keratohyalin granules, which are important for proper keratinocyte apoptosis [109]. It was hypothesized that anti-DFS70/LEDGFp75 autoantibodies may affect its pro-survival function and contribute to the development of skin disease [109]. However, this would be plausible only in the context of extracellular release of DFS70/LEDGFp75.

Takeichi et al. [103] observed that DFS70/LEDGFp75, in addition to inducing the IL-6/STAT-3 pathway in cultured skin cells, localized to the nucleus of keratinocytes in psoriatic lesions, which suggested a pivotal role for this autoantigen in protecting psoriatic keratinocytes under a stressful microenvironment. They suggested that downregulation of DFS70/LEDGFp75 may mitigate psoriasis symptoms by attenuating keratinocyte proliferation in psoriatic lesions.

Anti-DFS70/LEDGFp75 autoantibodies have also been found in 19.8 % of patients with alopecia areata (AA), an inflammatory skin condition that has autoimmune underpinnings, compared to 7.6 % of HI controls [21]. IHC analysis revealed that DFS70/LEDGFp75 localized to the outer root sheath cells of the hair follicle, the area that is targeted by the immune response in AA patients, suggesting that anti-DFS70/LEDGFp75 antibodies may contribute to AA pathophysiology [21].

### Anti-DFS70/LEDGFp75 autoantibodies in eye diseases

Anti-DFS70/LEDGFp75 antibodies have been detected in diverse eye diseases [9, 22, 25, 110]. Ayaki et al. [108, 111] reported that these antibodies induce cytotoxicity in LECs, suggesting a pathogenic role. They proposed that the antibodies absorb extracellularly released DFS70/LEDGFp75, preventing its re-entry into LECs where it acts as a pro-survival factor. This is consistent with previous observations that DFS70/LEDGFp75 is secreted by LECs and that its absorption by autoantibodies added to the culture medium reduced cell survival under stress [15].

Bizzaro et al. [22] reported that over 20 % of human sera showing the DFS-IIF pattern in HEp-2 cells also reacted strongly against reticular fibers of the lens and the corneal epithelium. These DFS-positive sera, however, produced different distribution patterns, suggesting the presence of companion autoantibodies targeting interacting ligands of DFS70/LEDGFp75. These authors noted that it is not easy to recognize the DFS pattern by IIF microscopy and therefore this method should not be used alone as the preferred technology to measure anti-DFS70/LEDGFp75 antibodies [22, 112].

Autoantibodies to DFS70/LEDGFp75 have also been reported in Vogt–Koyanagi–Harada (VKH) disease, an inflammatory disorder affecting multiple organs containing melanocytes, including uvea, skin, central nervous system, and inner ears [25]. Their presence was confirmed by ELISA in 67 % of VKH patients and in other patients with panuveitis, including sympathetic opthalmia, Behcet’s disease, and sarcoidosis (Table 3). Notably, these antibodies were also detected in 22 % of HI, which suggested the influence of background reactivity or selection of a low cutoff value in the ELISA.

Chin et al. [110] identified high-titer anti-DFS70/LEDGFp75 autoantibodies in three of six patients with atypical retinal degeneration. IHC studies using these autoantibodies demonstrated the presence of DFS70/LEDGFp75 in nuclei from murine retinal ganglion and pigment epithelial cells [110]. Consistent with this, DFS70/LEDGFp75 protected retinal pigment epithelial cells from nuclear damage induced by rhodopsin, a protein that forms nuclear aggregates causing cell death and retinal degeneration [113, 114].

### Anti-DFS70/LEDGFp75 autoantibodies in CFS

A low frequency of anti-DFS70/LEDGFp75 autoantibodies (3.3 %) was reported in adult patients with CFS [3]. However, other studies reported an elevated presence of these autoantibodies in children with CFS but not in children with fibromyalgia (FM) [115, 116]. These findings are intriguing and should be confirmed in large cohorts of adults and children with CFS and FM diagnoses. Children with other non-autoimmune conditions may also produce these autoantibodies, as highlighted by a recent case report of an 8-year-old patient with respiratory distress who presented high-titer anti-DFS70/LEDGFp75 autoantibodies with no evidence of SARD [117]. The authors concluded that these antibodies were a useful biomarker to rule out suspected autoimmune disease in that particular case [117]. It should be noted, however, that ANA prevalence, specificity, and titers may change during puberty, which could explain the observed differences in autoantibody frequencies between children and adults [118].

### Anti-DFS70/LEDGFp75 autoantibodies in cancer

During a screening of sera from patients with PCa for the presence of autoantibodies to tumor associated antigens, Daniels et al. [99] observed that the DFS-IIF pattern was predominant in sera from PCa patients compared to matched controls. Immunoblotting analysis of these DFS-IIF-positive sera, using PCa cell lysates as substrates, revealed that the majority reacted with a 70-kD protein band, and ELISA showed that 18.4 % of PCa sera reacted with this protein, compared to 5.5 % of controls. Overall, 22.3 % of the PCa sera reacted with DFS70/LEDGFp75 either by ELISA or immunoblotting, compared to 6.7 % of matched controls. Interestingly, the authors observed an incomplete correlation in the detection of these antibodies between the different immunoassays, which was attributed to differences in sensitivity and antigen conformation in the individual assays [99]. More recently, other groups have independently confirmed the presence of DFS70/LEDGFp75 autoantibodies in PCa sera [119–121]. These findings led to the hypothesis that DFS70/LEDGFp75 could be aberrantly expressed and functionally hyperactive in PCa and perhaps other human cancers [99]. Numerous studies have confirmed this hypothesis by showing altered DFS70/LEDGFp75 expression and function in various human cancer cell and tumor types, linking it to tumor aggressive properties [31, 34, 56–60, 72, 80–82, 86, 87, 90, 99, 122].

It should be noted, however, that other studies have reported low frequency of autoantibodies to DFS70/LEDGFp75 in cancer patients [22, 107]. It is not clear whether autoantibodies to DFS70/LEDGFp75 are more prevalent in PCa patients than in patients with other cancers. Therefore, it would be important to determine the frequency of these autoantibodies, using several detection platforms, in large cohorts of ethnically diverse patients with different cancer types as well as individuals at high risk of developing cancer.

### Low frequency of anti-DFS70/LEDGFp75 autoantibodies in SARD

The initial study on the clinical significance of these autoantibodies revealed a relatively low frequency (2–4 %) of these antibodies in patients with SARD [3]. This observation was later reproduced in more comprehensive studies performed by several other groups. For instance, Dellavance et al. [7] reported that 30 % of ANA-positive sera in a cohort of over 13,000 patients presented the DFS-IIF pattern, with IgG titers ranging from 1:80 to over 1:640. This was by far the overwhelming type of ANA-IIF pattern detected in this large unbiased sample cohort. Clinical information obtained for 81 of the DFS-IIF-positive serum donors indicated a diverse spectrum of disease conditions that included organ-based autoimmune diseases and inflammatory conditions. A key conclusion of this study was that anti-DFS70/LEDGFp75 autoantibodies are a relatively common finding among ANA-positive individuals with no evidence of SARD [7].

Muro and colleagues examined 500 SARD sera for the presence of anti-DFS70/LEDGFp75 antibodies, as well as for SARD-associated marker autoantibodies [28]. They found low frequencies of these autoantibodies and observed that 86 % of the SARD patients positive for anti-DFS70/LEDGFp75 autoantibodies also had at least one SARD-marker autoantibody. These authors concluded that patients with SARD producing anti-DFS70/LEDGFp75 antibodies as the only serum ANA-IIF pattern are rare and that such antibodies could be used as exclusion biomarkers of SARD in ANA-positive individuals.

Low prevalence (6.4 %) of these antibodies, detected by ELISA and immunoblotting, were also reported in a cohort of 103 Japanese patients with dermatomyositis (DM) [123]. Most patients producing these antibodies also produced DM-specific autoantibodies, including antibodies to MDA5, which are used as serological markers for aggressive disease, particularly complications with interstitial lung disease (ILD) [123, 124]. An interesting observation was that three DM-ILD patients producing both anti-DFS70/LEDGFp75 and anti-MDA5 antibodies who went into remission after therapy had decreased levels of anti-MDA5 autoantibodies concomitant with increased levels of anti-DFS70/LEDGFp75 antibodies [123]. However, a fourth patient with DM-ILD who produced both antibodies and succumbed to the disease showed unchanged levels of anti-MDA5 autoantibodies concomitant with decreased levels of anti-DFS70/LEDGFp75 antibodies. These observations raised the intriguing hypothesis, which needs to be further investigated in a larger patient cohort, that anti-DFS70/LEDGFp75 antibodies may serve a protective role [123]. However, it cannot be ruled out that these autoantibodies might be acting as sensors of DFS70/LEDGFp75 upregulation in response to the systemic stress produced by therapy in the surviving patients. To explore this possibility, it would be important to compare the circulating levels or diseased tissue expression of DFS70/LEDGFp75 in large cohorts of therapy-responding versus non-responding DM-ILD patients producing these antibodies.

### Anti-DFS70/LEDGFp75 autoantibodies in healthy individuals

Watanabe et al. [125] screened sera from 597 self-reported healthy hospital workers for the presence of ANAs and observed that 54 % of all ANA-positive individuals had anti-DFS70/LEDGFp75 antibodies. This led to the speculation that these antibodies may be naturally occurring in both apparently HI and diseased individuals. It should be cautioned, however, that hospital personnel tends to present higher ANA levels than blood donors or relatives of SARD patients [126].

Later studies confirmed that anti-DFS70/LEDGFp75 antibodies are more prevalent in apparently HI than in patients with SARD. For instance, Mariz et al. [26] screened 918 HI (negative history of SARD, infections, and inflammatory conditions) and 153 SARD patients for the prevalence of ANAs, as detected by HEp-2 IIF. The DFS-IIF and the nuclear fine speckled (unrelated to DFS70/LEDGFp75) patterns were the most frequent (approximately 33 and 46 %, respectively) in ANA-positive HI. Another similar HEp-2-IIF pattern characterized by fine grainy nuclear staining with staining of metaphase chromosomes, designated as quasi-homogeneous pattern, was observed in 4 % of the ANA-positive HI [26]. Confirmation that the DFS-IIF pattern was associated with antibodies to DFS70/LEDGFp75 was obtained by immunoblotting, whereas sera positive for the nuclear fine speckled and quasi-homogeneous patterns did not react with the protein. Interestingly, antibody titers reached 1:640 and 1:1280 in 50 % of the DFS-IIF-positive sera, with titers >1:5,120 in three individuals. Follow-up studies revealed that the presence and titers of anti-DFS70/LEDGFp75 antibodies were stable over the years and that the positive HI did not subsequently develop SARD or any evident disease [26]. This is in contrast to the known predictive value of disease marker ANAs for SARD diagnosis [127]. Mariz et al. [26] pointed to the difficulty of distinguishing the nuclear fine speckled and the quasi-homogeneous nuclear patterns from the DFS-IIF pattern in HEp-2 substrates, even by trained laboratory personnel, and recommended expanding efforts to address the reproducibility of ANA-HEp-2 test interpretations among different experts and commercial brands.

Mahler et al. [107] reported a prevalence of anti-DFS70/LEDGFp75 antibodies of 8.9 % (determined by DFS70-CIA) in a cohort of 124 serum samples from clinically defined HI with no history of SARD. This prevalence was significantly higher than in patients with SARD and non-SARD diseases, which exhibited prevalences below 6 %. These authors noted that in an SLE cohort there were no clinical differences between the few patients with anti-DFS70/LEDGFp75 antibodies and the patients without these antibodies, suggesting that the antibodies are not protective and do not correlate with disease activity. They also observed that in the SLE cohort all but one of the patients with anti-DFS70/LEDGFp75 antibodies also had other classical SLE-associated autoantibodies [107]. These findings reinforced the notion that anti-DFS70/LEDGFp75 antibodies are more prevalent in HI than in SARD patients. However, given that a small proportion (2–3 %) of SARD patients in this and other studies also produced these antibodies [3, 28], it cannot be asserted conclusively that these antibodies are highly accurate biomarkers for SARD exclusion, unless they are the only ANA specificity detected in the sera.

Studies with large cohorts of well-defined SARD patients are necessary to determine whether the presence of anti-DFS70/LEDGFp75 autoantibodies in these patients is coincidental or associated with a specific clinical phenotype or therapy. Along these lines, a novel immunoadsorption technology has been developed to increase the specificity of the ANA-HEp-2 cell assay [5]. Using recombinant DFS70/LEDGFp75 in the dilution buffer, anti-DFS70/LEDGFp75 antibodies are prevented from binding their target in HEp-2 cells [5, 6]. This then reveals the clinically relevant IIF pattern in sera with concomitant anti-DFS70/LEDGFp75 and other SARD-marker autoantibodies.

### Prevalence of anti-DFS70/LEDGFp75 antibodies in routine ANA testing

Mahler et al. [107] screened 3,263 serum samples submitted for ANA testing for the presence of anti-DFS70/LEDGFp75 autoantibodies and observed that 1.62 % presented the DFS-IIF pattern, which was confirmed to correspond to anti-DFS70/LEDGFp75 autoantibodies when evaluated by DFS70-specific ELISA and CIA. Bizzaro et al. [22] also observed low frequency (0.8 %) of sera displaying the DFS-IIF pattern in HEp-2 cells in 21,512 samples screened for ANA in the clinical laboratory. Two additional studies also reported low frequencies (<4 %) of this pattern in thousands of sera screened for ANAs [128, 129].

Miyara et al. [24] evaluated the clinical value of anti-DFS70/LEDGFp75 autoantibodies in patients undergoing routine ANA testing. Analysis of sera from 100 consecutive patients with DFS-IIF pattern and 100 patients with other patterns, using the ANA-HEp-2 test, DFS70 CIA, and QUANTA Lite ANA Screen ELISA (which simultaneously detects serum autoantibodies to common SARD-related autoantigens), revealed that only 5.5 % of patients with anti-DFS70/LEDGFp75 antibodies had SARD. Most of the anti-DFS70/LEDGFp75 antibody-positive samples were negative on the ANA Screen ELISA. When combining a negative ANA ELISA result with a positive anti-DFS70/LEDGFp75 antibody test result, good discrimination between SARD and non-SARD patients was obtained [24], strengthening the notion that when found as the only ANA-IIF specificity in patient serum this antibody could serve as a reliable exclusion marker of SARD.

Fitch-Rogalsky et al. [130] analyzed the clinical and serological features of patients referred through a rheumatology central triage system because of a positive ANA test. Of 15,357 referred patients, 4.1 % had positive ANA. The frequency of the anti-DFS70/LEDGFp75 autoantibody in 225 archived sera from the patients evaluated by a rheumatologist was 15.1 %, and this was the sole autoantibody in 70.6 % of the anti-DFS70/LEDGFp75-positive patient sub-cohort. Among the anti-DFS70/LEDGFp75-positive patients, 6 % had SARD with other autoantibodies. This reinforced the notion that when these autoantibodies are present in patients with SARD they usually coexist with other disease marker autoantibodies and tend to exclude a SARD diagnosis when they are the sole ANA-IIF specificity in human sera. The detection of anti-DFS70/LEDGFp75 antibodies is now used in this triage system to help prioritize patients for referral and thereby reducing waiting times for urgent cases.

## Consequences of anti-DFS70/LEDGFp75 antibodies for ANA testing

Accurately identifying the DFS-IIF pattern by ANA-HEp-2 screening is not an easy task [22, 26, 107, 112, 131]. Bizzaro et al. [22, 112] noted that 86 % of moderate to high-titer sera producing the DFS-IIF pattern in HEp-2 slides failed to recognize DFS70/LEDGFp75 in DFS70-specific ELISA systems. In addition, analysis of these sera in HEp-2 slides from various commercial sources gave inconsistent results. These investigators attributed these discrepancies to different HEp-2 substrate preparations, the type of DFS70/LEDGFp75 epitope exposed in the ELISA systems, and the identification of the DFS-IIF pattern by non-expert clinical interpreters.

These concerns highlight the importance of accurately identifying the anti-DFS70/LEDGFp75 antibodies using a combination of detection methods that may include, in addition to ANA-HEp-2, immunoblotting, DFS70-CIA, and ELISA-DFS70, as well as expert interpretation of these assays. The use of recombinant DFS70/LEDGFp75 peptides encompassing the autoepitope region for autoantibody immunoadsorption is critical to validate the results [5, 6]. Fritzler [131] argued that inter-laboratory discrepancies regarding detection of anti-DFS70/LEDGFp75 autoantibodies during routine ANA screening could be rendered moot by the availability of a second, validated test that complements the ANA results. While this would be ideal, the best practice for routine diagnostics would be a single, well-characterized assay that has been widely validated in various international laboratories.

Another consideration is the possibility that nuclear autoantigens other than DFS70/LEDGFp75 may also produce the DFS-IIF pattern. DFS70/LEDGFp75 is a component of nucleoprotein complexes associated with transcription regulation, and some of its interacting partners co-localize with this protein, producing an identical DFS-IIF pattern [42, 52–55].

Since ANAs are generally considered reliable biomarkers for SARD and are included in the classification criteria for SLE [132], ANA–HEp-2 testing outside a proper clinical framework may yield a sizable portion of ANA-positive individuals with no consistent evidence of SARD [133]. This could cause undue concern and anxiety in patients, their families and physicians alike, and even lead to unwarranted therapeutic interventions [26, 133–135]. This becomes even more crucial with compelling evidence that autoantibodies may precede the clinical onset of SARD by many years [127]. Not all sera demonstrating the DFS-IIF pattern are from HI and it remains unclear whether this staining pattern is universally recognized in clinical diagnostic laboratories. The discrimination between DFS-IIF pattern and the “quasi-homogeneous pattern” might be challenging in routine diagnostic laboratories [131]. This underlines the importance of a better understanding of anti-DFS70/LEDGFp75 antibodies and the inclusion of testing for these antibodies into diagnostic algorithms [5, 6]. Sera with the DFS-IIF pattern should be tested for anti-DFS70/LEDGFp75 antibodies using a specific immunoassay and then the test results and their significance clearly explained to patients [24].

## Potential impact of anti-DFS70/LEDGFp75 antibodies on SLE classification criteria

A positive ANA test is part of the SLE criteria developed by the American College of Rheumatology (ACR) and the Systemic Lupus International Collaborating Clinics (SLICC) [136, 137]. However, since anti-DFS70/LEDGFp75 antibodies are not associated with SLE and rarely found in isolation in SLE patients [3, 24, 26, 28, 107] and could be confused with other ANA-IIF patterns [26], these antibodies might reduce the specificity of the criteria. Therefore, consideration should be given to the concept that anti-DFS70/LEDGFp75 antibodies, when present as the only ANA-IIF pattern in serum, could serve as an exclusion criterion in the diagnosis and classification of SLE. Thus, a revised ACR criterion #11 might state: “An abnormal titer of antinuclear antibody by immunofluorescence, *excluding monospecific anti*-*DFS70/LEDGFp75 reactivity*, or an equivalent assay at any point in time and in the absence of drugs.”

## What exactly are the anti-DFS70/LEDGFp75 autoantibodies trying to tell us?

### Are these autoantibodies natural and protective?

Natural autoantibodies, both IgM and IgG, play a critical, protective role by assisting in the clearance or neutralization of apoptotic cell debris, which is essential to prevent the release of intracellular self-antigens and danger signals that could induce inflammatory and autoimmune responses [137–140]. To date there is little objective and formal evidence that anti-DFS70/LEDGFp75 autoantibodies are natural antibodies playing protective roles. While their low frequency in SARD, presence in 5–10 % of HI who do not develop autoimmune conditions after years of follow-up, and increased levels in DM-ILD patients who went into remission after therapy, suggest the possibility that they could play a protective role, further studies are warranted to support this role. These autoantibodies might function in the removal of DFS70/LEDGFp75 cleavage fragments from debris generated during cell death associated with tissue damage. This would not only attenuate local inflammatory responses, but also prevent these fragments from enhancing cell death [32].

### Are these autoantibodies pathogenic?

The only evidence that these autoantibodies could play pathogenic roles comes from the studies by Ayaki et al. [108, 111] reporting their cytotoxicity in vitro against LEC and cultured lens organs. In this context, when upregulated and activated by stress, DFS70/LEDGFp75 could be released into the extracellular milieu and uptaken by cells in the local tissue microenvironment where it may transcriptionally activate stress response and pro-inflammatory pathways. Ayaki et al. [108, 111] suggested that binding of the autoantibodies to released DFS70/LEDGFp75 exerts a pathogenic role by preventing its uptake by neighboring cells.

### Are these autoantibodies sensors of microenvironmental stress and inflammation?

The broad spectrum of diseases and conditions associated with the presence of anti-DFS70/LEDGFp75 autoantibodies (Table 3) points to an augmented state of cellular oxidative stress, local inflammation, and tissue damage (i.e., bladder, eye, skin, prostate), as potential common denominators. Dying cells, which in vivo can be derived from tissue damage, are a source of intracellular autoantigens that are clustered in apoptotic blebs or post-translationally modified [141–143]. Defects in the clearance of dying cells in certain autoimmune diseases or inflammatory conditions, associated with inflammatory necrosis or progression of apoptosis to secondary necrosis, could lead to a pro-inflammatory environment, thus facilitating autoantibody responses to aberrantly modified autoantigens [144]. Primary and secondary necrosis, and necroptosis, also yield unique autoantigen cleavage fragments, generated by lysosomal cathepsins that are recognized by autoantibodies [98, 145, 146].

**Fig. 4:**
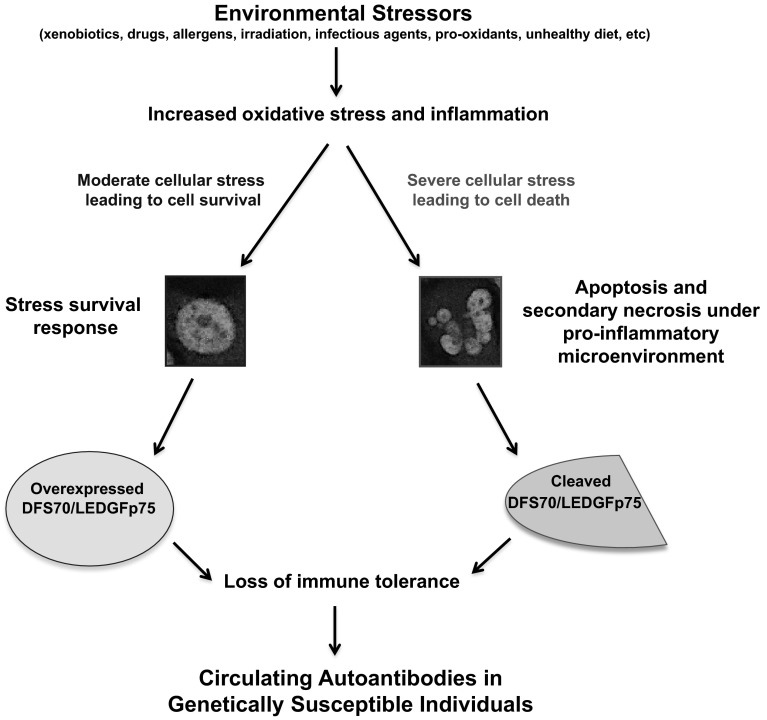
Model for the elicitation and role of anti-DFS70/LEDGFp75 autoantibodies. Environmental stressors may induce oxidative stress and inflammation in certain tissues, leading to a stress response characterized by DFS70/LEDGFp75 upregulation and activation. Overexpression of DFS70/LEDGFp75 during a moderate cellular stress response or its apoptotic cleavage under tissue damage and inflammation induced by severe stress may alter its immunogenicity, leading to the elicitation of autoantibodies in genetically susceptible individuals

As mentioned previously, DFS70/LEDGFp75 is cleaved during apoptosis into fragments that are recognized by human autoantibodies and that persist during secondary necrosis [32, 33, 98, 99]. Its overexpression in disease-affected tissues, combined with its proteolytic cleavage or involvement in stress-induced protein complexes that influence its processing by the immune system, may alter its immunogenicity in a pro-inflammatory microenvironment, making it a target of autoantibodies (Fig. 4). There is evidence that tissue overexpression, mutation, or posttranslational modification of intracellular autoantigens in a pro-inflammatory context may trigger the elicitation of autoantibodies [143, 145–147]. It is then plausible that autoantibodies to DFS70/LEDGFp75 could then be considered as “sensors” of microenvironmental stressors associated with inflammation, tissue damage, and altered expression of this protein.

## Conclusions

The answer to the question of what are the anti-DFS70/LEDGFp75 autoantibodies trying to tell us, first posed by our group a decade ago [8], still eludes the field of ANA research. However, our current knowledge of the DFS70/LEDGFp75 autoantigen-autoantibody system, reviewed above, provides the following clues, which could help us understand its biological and clinical significance:

1. DFS70/LEDGFp75 is a stress response transcription co-activator that contributes to the upregulation of stress protective and inflammatory genes, leading to cellular survival under environmental stress in both health and disease contexts.
2. Altered function, expression, or structure of DFS70/LEDGFp75 in a microenvironment characterized by inflammation and tissue damage may contribute to disease pathogenesis and autoantibody elicitation.
3. Autoantibodies to DFS70/LEDGFp75 preferentially target a functionally important and conserved region in its C-terminal domain.
4. Anti-DFS70/LEDGFp75 IgG autoantibodies can be found at high titers in apparently healthy individuals and in patients with diverse, non-SARD, inflammatory conditions.
5. When present in patients with SARD, anti-DFS70/LEDGFp75 autoantibodies are usually accompanied by SARD-marker antibodies.
6. Anti-DFS70/LEDGFp75 autoantibodies may serve as exclusion markers of SARD when present as the only ANA specificity in patient sera.
7. Detection of anti-DFS70/LEDGFp75 autoantibodies by ANA-HEp-2 IIF test is not always reliable, and positive tests should be confirmed using other detection platforms.

**Fig. 5:**
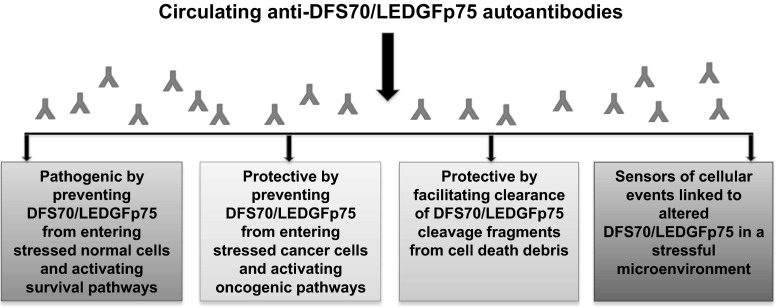
Potential roles of anti-DFS70/LEDGFp75 autoantibodies. Depending on the context in which they arise, these autoantibodies could play pathogenic, protective, or sensor roles

We propose that depending on the context, anti-DFS70/LEDGFp75 autoantibodies could serve as protective antibodies with no specific disease relevance, pathogenic antibodies in certain conditions, or sensors of increased stress and inflammatory cellular damage in the local microenvironment of the affected organ or tissue (Fig. 5). It cannot be ruled out that the presence of these autoantibodies could be an “epiphenomenon” unrelated to the disease conditions listed in Table 3, and coincident as the result of yet to be identified factors or co-morbid conditions. The relatively low frequency (<15 %) of these antibodies in most HI and patient cohorts evaluated for their presence indicates that only certain individuals produce them, which points to genetic susceptibility in their generation.

We recommend that comprehensive information on the health history, lifestyle, ethnicity, geographic location, and exposure to environmental stressors or xenobiotics should be acquired for both HI and patients producing anti-DFS70/LEDGFp75 autoantibodies. Initiatives such as the CARTaGENE biobank study [148], which seeks to identify genetic and environmental factors associated with disease-related quantitative traits, might help determine whether these antibodies could be linked to specific geographic areas and/or exposures that may influence the levels of oxidative stress in a particular tissue microenvironment, leading to aberrant DFS70/LEDGFp75 expression and autoantibody production. We anticipate that as our knowledge of the DFS70/LEDGFp75 autoantigen-autoantibody system advances, its elusive biological and clinical significance will unravel, leading to translational applications.

## Abbreviations

AA: Alopecia areata
AD: Atopic dermatitis
AIDS: Acquired immunodeficiency syndrome
ANAs: Antinuclear autoantibodies
CFS: Chronic fatigue syndrome
DFS: Dense fine speckles
DFS70: Dense fine speckled autoantigen of 70 kD
DFS70-CIA: DFS70 chemiluminescence assay
DM: Dermatomyositis
ELISA: Enzyme-linked immunosorbent assay
FM: Fibromyalgia
HDGF: Hepatoma derived growth factor
HI: Healthy individuals
HIV: Human immunodeficiency virus
HIV-IN: Human immunodeficiency virus integrase
HLA: Human leukocyte antigen
HRP-2: HDGF-related protein 2
IBD: Integrase-binding domain
IC: Interstitial cystitis
ILD: Interstitial lung disease
IHC: Immunohistochemistry
IIF: Indirect immunofluorescence microscopy
LEC: Lens epithelium cells
LEDGFp75: Lens epithelium-derived growth factor protein of 75 kD
LMP: Lysosomal membrane permeabilization
miRNA: Micro RNA
MLL: Mixed leukemia lineage
PCa: Prostate cancer
PSIP1: PC4 and SFRS1 interacting protein 1
PWWP: Proline-tryptophan-tryptophan-proline motif
SARD: Systemic autoimmune rheumatic disease
SFRS1: Serine/arginine-rich splicing factor 1
SLE: Systemic lupus erythematosus
UVB: Ultraviolet B
VKH: Vogt–Koyanagi–Harada
